# Raised serum creatinine at admission to critical care is independently associated with mortality in patients with decompensated alcoholic liver disease

**DOI:** 10.1186/cc9518

**Published:** 2011-03-11

**Authors:** A Whiteside, P Whiting

**Affiliations:** 1 Sheffield Teaching Hospitals NHS Trust, York, UK; 2 Northern General Hospital, Sheffield, UK

## Introduction

Patients with decompensated alcoholic liver disease have a high mortality if they require critical care. Previous studies have indicated that patients who required renal replacement therapy have high mortality, but there is little research on the mortality rate of those with renal impairment not requiring support.

## Methods

A retrospective cohort study of patients with a diagnosis of decompensated alcoholic liver disease admitted to the critical care department of two hospitals over a 3-year period was conducted (*n *= 51).

## Results

There was no significant difference in the ages (50.8 and 50.3, *P *= 0.9) or sexes of those who survived and those who died during hospital stay. Hospital, 6-month and 1-year mortality rates were 45%, 49% and 51%, respectively. There was no significant difference in the number of patients requiring advanced respiratory support (69% vs. 74%, *P *= 0.76). Ninety-four per cent of patients who had a serum creatinine of 150 mmol/l or greater at admission to critical care died during their hospital stay.

## Conclusions

The futility of admitting patients with decompensated alcoholic liver disease with serum creatinine of 150 mmol/l or greater should be considered at the time of referral to critical care, as they have a 94% mortality.
